# Wild Boar-Related Injuries in Wilderness Environments: Field-Oriented Management Principles and Evidence-Informed Clinical Recommendations

**DOI:** 10.3390/jcm15197381

**Published:** 2026-09-23

**Authors:** Markus Maier, Leonard P. N. Maier, Simon Hackl, Christoph Schmitz, Nicola Maffulli

**Affiliations:** 1 Department of Orthopedics and Trauma Surgery, Starnberg Orthopedics Clinic, 82319 Starnberg, Germany; 2 Department of Orthopaedics and Trauma Surgery, LMU University Hospital, Ludwig-Maximilians-University of Munich, 81377 Munich, Germany; 3 Department of Orthopaedics and Trauma Surgery, Klinikum Nordfriesland, 25813 Husum, Germany; lpn.maier@gmail.com; 4 Department of Trauma Surgery, BG Unfallklinik Murnau, 82418 Murnau, Germany; simon.hackl@bgu-murnau.de; 5 Chair of Neuroanatomy, Institute of Anatomy, Faculty of Medicine, LMU Munich, 80336 Munich, Germany; christoph_schmitz@med.uni-muenchen.de; 6 Department of Trauma and Orthopaedic Surgery, Faculty of Medicine and Psychology, University La Sapienza, 00189 Roma, Italy; n.maffulli@qmul.ac.uk; 7 School of Pharmacy and Bioengineering, Keele University Faculty of Medicine, Stoke on Trent ST5 5BG, UK; 8 Centre for Sports and Exercise Medicine, Barts and the London School of Medicine and Dentistry, Mile End Hospital, Queen Mary University of London, London E1 4NS, UK

**Keywords:** wild boar injury, wilderness trauma, penetrating extremity wounds, austere environment medicine, trauma management algorithm

## Abstract

Wild boar-related trauma is uncommon but may result in severe injuries that pose diagnostic and therapeutic challenges, particularly in wilderness or resource-limited environments. Most scientific literature consists of isolated case reports, regional series, and forensic descriptions; clinical guidance is therefore fragmented. This study synthesizes two complementary evidence sources: a structured observational cohort documenting 101 injuries sustained by boar hunters during organized hunts in Germany, and a comprehensive systematic review of all globally published wild boar- and feral pig-related human injuries. The observational cohort provided internally consistent epidemiologic information on who was injured, under what circumstances, how quickly assistance was obtained, and how injuries were treated and ultimately healed. The systematic review added detailed insight into injury morphology and severity across diverse settings, including reports of deep wound tracts, extensive soft-tissue disruption, significant contamination, and the risk of hemorrhage, neurovascular compromise, or thoracoabdominal penetration. The systematic review was conducted in accordance with PRISMA 2020 principles and included explicit study identification, screening, and eligibility assessment. Integrating these sources enabled a field-oriented approach to assessment and management, emphasizing early hemorrhage control, broad-spectrum antibiotic therapy, appropriate use of imaging, thorough surgical exploration when indicated, and region-specific tetanus or rabies prophylaxis. Together, these findings support clinicians working in remote or austere environments who may encounter these rare but potentially serious injuries.

## 1. Introduction

Wild boar (*Sus scrofa*) populations continue to rise across Europe and many other regions, driven by favorable climatic conditions, landscape fragmentation, agricultural food availability, and reduced predator pressure [1,2,3,4]. As populations expand, human–wild boar contact has increased across rural, peri-urban, and even urban areas, resulting in property damage, traffic collisions, zoonotic disease risks and, on occasion, traumatic injuries [1,4]. Wild boar attacks make up only a small proportion of wildlife-related trauma, but the resulting injuries can be severe. Solitary adult males are disproportionately associated with high-energy encounters given their size, behavior, and tusk morphology [5,6].

The published clinical and forensic literature contains numerous reports of penetrating or tearing wounds, most often affecting the lower extremities, groin, or trunk, and typically caused by close-range charges [5,7,8]. These accounts describe deep and irregular wound tracts, extensive soft-tissue disruption, and heavy contamination with soil, vegetation, or organic material, sometimes accompanied by complex polymicrobial infection [4,9]. Tusk-inflicted injuries may involve sharp-force penetration with limited blunt components, with vascular or visceral injury even when the external wound appears deceptively small [7,8]. Inadequate debridement or delayed recognition of contamination has been associated with secondary systemic complications such as sepsis [10]. Together, these reports highlight the potential severity of wild boar-related trauma across a diverse range of agricultural, wilderness, and peri-urban contexts.

Despite these recurring patterns, the literature has remained fragmented, consisting predominantly of isolated case reports and small series without a unified clinical framework. This fragmentation complicates decision-making for prehospital providers, wilderness responders, and clinicians who may encounter these rare injuries in remote or resource-limited settings.

Wild boar-related trauma and explosion injuries share no common pathophysiology or injury mechanics, but both represent rare and potentially life-threatening events that many clinicians may encounter only once or a few times over their entire career. In this regard, the situation parallels recently published guidelines for the management of explosion trauma, which emphasize the importance of structured, evidence-based pathways for uncommon but high-stakes injury patterns [11,12]. These guidelines demonstrate how standardization can support rapid, high-quality decision-making when prior personal experience is inherently limited.

Comparable examples from the wilderness-medicine domain further underscore the value of formalized guidance for infrequent critical events. The Wilderness Medical Society has issued comprehensive clinical practice guidelines for scenarios such as drowning [13] and for the medical direction of search-and-rescue teams [14]. These documents highlight the essential role of structured recommendations in austere and prehospital environments, where limited resources, prolonged extraction times, and variable provider experience may make administering clinical care more difficult. The present study follows the same conceptual rationale: to provide a consistent, field-oriented management framework for a rare but potentially severe injury mechanism.

To address long-standing gaps in the literature, the present study integrates two complementary sources of evidence originating from prior work [15]. The first is a structured observational cohort of hunters who sustained wild boar-related injuries during organized hunting activities in Germany [15]. The second is a comprehensive systematic review, summarizing all globally published clinical, forensic, and epidemiologic accounts of injuries inflicted by wild boars and feral pigs. While the earlier study [15] focused primarily on dataset generation, epidemiologic characterization, and validation of these two evidence sources, the present study builds upon these previously developed datasets to provide a dedicated clinical synthesis focused on practical assessment, management principles, and prevention strategies in austere environments. The current manuscript therefore does not present new primary data, but rather translates previously established datasets into a structured clinical decision-support framework for rare but potentially severe injuries.

## 2. Materials and Methods

This study is derived from two complementary evidence sources developed in previous work [15]. The first is a structured observational cohort of hunters who sustained wild boar-related injuries during organized hunting activities in Germany [15]. The second is a systematic review of the international literature on human injuries inflicted by wild boars or domestic swine [15]. Together, these sources provide both consistent, context-specific data and a broader global perspective on the clinical characteristics and management of such injuries. As both datasets had already been fully completed prior to development of the present study, no additional primary data collection was performed for the current study.

The observational cohort included 101 hunters who sustained wild boar-related trauma in the context of licensed hunting activities [15]. Data were collected through standardized, physician-led telephone interviews. For each participant, the interviews documented demographic characteristics, hunting experience, situational details of the incident, characteristics of the offending animal, the time to first help, the subsequent treatment setting, the use of operative or non-operative management, rehabilitation requirements, final healing outcomes, work absenteeism, and post-injury behavioral adaptations. Injuries were categorized into three predefined groups: closed injuries, open injuries treated on an outpatient basis, and open injuries requiring inpatient care. This framework reflected treatment pathways and overall outcomes but did not capture detailed clinical descriptors such as wound morphology, imaging findings, or intraoperative observations.

Within this cohort, four participants provided substantially more detailed accounts of their injuries and clinical course. These narratives were obtained within the same interview framework and represent a more elaborate subset of the existing cohort rather than additional cases. Although they cannot be generalized to the full sample, they illustrate the range of complications that can occur after wild boar-related trauma.

In the first case, a hunter between 20 and 30 years old sustained an approximately 10 cm open wound on the medial aspect of the lower leg during a tracking operation. The wound extended to the muscle fascia, and the animal was killed at the scene. The patient reached hospital within one hour and underwent outpatient wound cleansing and primary closure without drainage; postoperative antibiotic therapy was not initiated. After seven days, wound-edge necrosis and a subcutaneous abscess developed beneath the suture line, necessitating inpatient revision with debridement, jet lavage, irrigation, negative-pressure wound therapy, and a planned second-look procedure with further irrigation and secondary closure. The wound subsequently healed without long-term impairment.

The second case involved a hunter between 50 and 60 years old who sustained an approximately 15-centimeter-long laceration on the medial aspect of the lower leg, extending to the periosteum of the tibia, also during a tracking operation. Hospital presentation occurred within six hours after injury. Initial management consisted of wound irrigation and primary closure on an outpatient basis, again without systemic antibiotic therapy. Within two days, a deep wound infection developed, and the patient required inpatient revision surgery and open wound treatment. The clinical course was prolonged but ultimately resulted in complete healing.

In the third case, a hunter between 30 and 40 years old suffered an extensive medial lower-leg wound extending from the ankle to the knee, with a reported depth “down to the bone”, during tracking. The patient presented to hospital within one hour and received outpatient wound irrigation and primary closure. In the following days, clinical condition deteriorated with signs of sepsis, and inpatient surgical revision became necessary. The subsequent treatment included extensive debridement, fasciotomy for compartment syndrome, vascular repair, and open wound management. The inpatient stay lasted 21 days, and the patient ultimately recovered fully.

The fourth case concerned a hunter between 20 and 30 years old with an approximately 12 cm open lateral thigh wound reaching the femur, again incurred during a tracking operation. Hospital presentation took place within one hour. Initial management consisted of primary closure during a short inpatient stay. Six weeks later, persistent local inflammation prompted operative revision, during which a retained wooden foreign body was identified and removed from the wound base. After this procedure, the wound healed completely.

These four cases were not included in quantitative analyses but were used qualitatively to illustrate potential postoperative complications and the impact of initial management decisions, particularly in relation to primary closure and the recognition of deep contamination or retained foreign material.

The second evidence source was a systematic review [15] conducted in accordance with the 2020 PRISMA (Preferred Reporting Items for Systematic Reviews and Meta-Analyses) guidelines [16], and which included all records indexed in PubMed and Ovid/Embase from inception through 12 February 2026. To improve methodological transparency in the present study, the corresponding PRISMA flow diagram from the underlying systematic review [15] is included as Figure 1, and the complete search strategy is shown in Table 1.

Eligible studies included clinical reports, case series, retrospective studies, forensic reports, and epidemiologic studies describing human injuries directly caused by wild boars, feral pigs, or domestic swine when clinically relevant injury mechanisms overlapped with wild boar trauma. Studies were included irrespective of geographic origin. Studies were excluded if they did not describe human injury events, focused exclusively on veterinary topics, were duplicate datasets, described non-traumatic animal encounters, or lacked sufficient clinical detail for meaningful interpretation.

Titles, abstracts, and full texts were screened in a multistage process. Duplicate records were removed prior to screening. Records identified through database searches, reference screening, and additional manual searches were assessed for eligibility as described in the PRISMA flow diagram (Figure 1). After multistage screening, 41 studies [4,17,18,19,20,21,22,23,24,25,26,27,28,29,30,31,32,33,34,35,36,37,38,39,40,41,42,43,44,45,46,47,48,49,50,51,52,53,54,55,56] were included. These reports originated from a broad range of settings, including agricultural, rural, peri-urban, wilderness, and subsistence environments, and described a wide variety of injury mechanisms, anatomical patterns, and management strategies. Table 2 provides an overview of the 41 included studies [4,17,18,19,20,21,22,23,24,25,26,27,28,29,30,31,32,33,34,35,36,37,38,39,40,41,42,43,44,45,46,47,48,49,50,51,52,53,54,55,56] and the contextual literature [1,2,3,5,6,7,9] used to frame the international burden and variability of wild boar-related injuries, while detailed study characteristics are presented in Table A1 in Appendix A.

As the available literature consisted predominantly of case reports, small case series, retrospective analyses, and forensic reports with substantial methodological heterogeneity, formal meta-analysis was not feasible. A structured qualitative appraisal was therefore performed in [15] by assessing study design, clinical detail, completeness of outcome reporting, and relevance to acute trauma management. Individual case reports were used primarily to illustrate uncommon injury mechanisms, complications, or management challenges rather than to generate independent high-level recommendations.

A hierarchical synthesis approach was applied. The observational hunters cohort [15] served as the primary empirical foundation because of its consistent methodology and internal coherence. The systematic review [15] was then used to broaden the clinical perspective, provide additional context, and highlight rare injury mechanisms or management challenges that could not be captured within the observational hunters cohort [15] alone. Individual case reports identified through the systematic review [15] were incorporated narratively when they complemented, but did not outweigh, the structured observational data. The observational hunters cohort [15] did not include systematic information on wound morphology, imaging results, physiological responses, or operative details; therefore, these aspects were informed either by the systematic review [15] or by the four detailed case narratives.

Based on this hierarchical evidence synthesis, the final management recommendations presented in this study were developed as evidence-informed clinical recommendations. No formal guideline development process, consensus procedure, or external recommendation grading system was performed.

The observational hunters cohort [15] study received approval from the Ethics Committee of Ludwig-Maximilians-University of Munich (Munich, Germany; reference 24-0377; approval date 5 June 2024). All procedures were conducted in accordance with General Data Protection Regulation (GDPR), and written informed consent was obtained from all participants prior to the telephone interviews. Participants first received written study information and consent documents and subsequently completed structured physician-led telephone interviews only after providing documented written consent. The systematic review [15] relied exclusively on previously published data and did not require additional ethical approval.

## 3. Results

### 3.1. Epidemiology

The previously established observational hunters cohort [15] included 101 injured hunters, all of whom had sustained wild boar-related trauma during organized hunting activities in Germany. Participants were experienced hunters with 24–31 years of hunting experience across injury categories. Most incidents occurred during tracking operations, which accounted for the majority of closed injuries (18 of 23; 78.3%), outpatient open injuries (42 of 46; 91.3%), and inpatient open injuries (24 of 32; 75.0%). Events took place predominantly in daylight, and male wild boar were responsible for most injuries, with their proportion increasing in the more severe injury categories. Injuries were concentrated in the autumn–winter hunting season, consistent with the timing of driven hunts. Because the dataset [15] was limited to hunting situations, it could not provide conclusions about risks in non-hunting environments. Qualitative remarks from participants described the encounters—particularly during tracking—as sudden and difficult to avoid. Many participants emphasized the importance of daylight tracking, cooperation within the hunting party, and the value of dogs and protective equipment. Notably, none of the hunters stopped hunting after the event [15].

Overall, the cohort outlined a clear epidemiologic context: experienced hunters, mainly injured during daytime tracking of wild boar, most often by male animals and largely within the main hunting season [15].

The systematic review [15] added substantial global epidemiologic breadth to these structured observations, and demonstrated that comparable patterns arise in a wide range of environments far beyond recreational hunting. Numerous international reports described injuries among farmers tending crops, forest workers managing timber or underbrush, hikers traversing wooded paths, and individuals engaging in routine rural activities such as collecting firewood, searching for truffles, or walking near water sources where boars may forage [38,39,40,41,42,43,44,45,46,47,48,49,50]. In several countries, particularly in parts of southern and eastern Asia, injuries occurred when humans inadvertently entered the resting areas of boar sounders or disturbed lone males during feeding [30,37,38]. Reports from the Mediterranean region and Japan additionally highlighted peri-urban incidents in which expanding wild boar populations moved into suburban gardens, parks, and garbage-rich environments, increasing human exposure even among individuals without any direct wildlife-related occupation [4,34,37].

Seasonal variation is another important epidemiologic dimension. In the original observational hunters cohort [15], most injuries clustered during autumn and winter driven hunts, reflecting hunting calendars and seasonal shifts in boar behavior. In contrast, the systematic review [15] described incidents occurring year-round, with peaks in spring and late summer linked to breeding behavior, juvenile dispersal, and shifts in food availability. Regarding injury causation, male boars predominated in both evidence sources, which aligns with known behavioral ecology: adult males tend to defend themselves more aggressively, display less flight behavior when confronted, and possess larger, sharper tusks capable of producing deeper wound tracts upon impact [15].

Population dynamics further affect exposure frequency. Rising wild boar numbers in Europe, driven by abundant agricultural forage, warmer winters, and reduced natural predation, have increased contact with humans [1,2,4]. Similar demographic expansion has occurred in Japan and parts of South Asia, where land-use change, depopulation of rural settlements, and the abandonment of hillside farmland have drawn animals into previously low-exposure areas [30,34,37]. These ecological changes, combined with the adaptability and high reproductive rate of the species, indicate that wild boar encounters may increase in frequency in coming years.

Although fatalities are rare relative to incidence, the systematic review [15] documented several deaths resulting from catastrophic vascular injury, severe thoracoabdominal penetration, or delayed recognition of internal damage. Such outcomes underscore that, even though most encounters are survivable when managed promptly, the potential for life-threatening trauma is present in all settings where humans and wild boars interact. These findings additionally support the importance of preventive education, situational awareness, and emergency preparedness in both hunting and non-hunting populations at risk of wild boar encounters. Together, the structured patterns identified in the observational hunters cohort [15] and the globally diverse reports summarized in the systematic review [15] form a coherent epidemiologic profile of a multifactorial and geographically widespread injury mechanism.

### 3.2. Clinical Presentation

The structured dataset of the original observational hunters cohort [15] contained only limited information about the acute clinical presentation of injuries. It distinguished cases by treatment pathway (closed injuries, open injuries treated on an outpatient basis, and open injuries requiring inpatient care) but did not include systematic descriptions of wound appearance, severity, bleeding, pain, neurovascular status, or physiological parameters. From the predefined treatment categories, it was evident that presentations ranged from minor closed trauma to open wounds requiring hospital admission and surgery. However, because the dataset did not record specific clinical findings at the time of presentation, these categories reflect treatment pathways rather than detailed injury morphology or physiological status.

A small subset of four participants provided more elaborate qualitative accounts describing delayed infections, wound necrosis, abscess formation, sepsis, compartment syndrome and, in one instance, the removal of a retained wooden foreign body during late revision surgery. These narratives illustrate the potential complexity of individual postoperative courses, but they cannot be extrapolated to the cohort as a whole. Overall, the dataset of the original observational hunters cohort [15] offered structured information concerning who was injured and how the injuries were managed, but only limited insight into the immediate clinical appearance or severity of the injuries at presentation.

The systematic review [15] supported and expanded these findings, demonstrating that similar clinical patterns appeared consistently across international case reports. Penetrating lower-extremity injuries were the hallmark presentation worldwide, often accompanied by extensive skin flaps, deep gashes, and narrow subcutaneous tracts that extended far beyond the visible entry point [4,36,49]. This pattern is consistent with the curved upward trajectory of wild boar tusks, which may create deceptively small external wounds while causing extensive deeper tissue disruption. These tracts often contained embedded debris such as soil, vegetation, hair, or fragments of clothing, all of which significantly elevate the risk of infection [4,9,46]. Thoracic injuries described in the systematic review [15] frequently presented with chest pain, dyspnea, subcutaneous emphysema, or altered breath sounds [4,23,36]. Pneumothorax and hemothorax may be occult in the early stages but progress quickly and require urgent decompression. Abdominal injuries often exhibited localized tenderness, distension, or guarding, but several case reports highlighted initial minimal symptoms despite significant internal damage, including bowel perforation, mesenteric bleeding, or diaphragmatic tears [34,42,50].

Upper-extremity and hand injuries, although less common in the observational hunters cohort [15], were well described in the systematic review [15]. These often involved defensive wounds sustained when victims attempted to push the animal away or shield themselves [4,37,44]. Such injuries included tendon lacerations, neurovascular compromise, or deep-space involvement of the palm or forearm [4,24,29]. Head and neck injuries were rare but were associated with exceptionally high morbidity [4,5,29]. Case reports from multiple countries described facial lacerations, orbital penetration, and blunt head trauma resulting from charges at close range or from individuals being knocked to the ground and trampled [5,50,52].

Pain severity was consistently reported across both evidence sources [15]. Many patients required significant analgesia early in their evaluation to allow adequate inspection of the wound [4,34,37]. The combination of pain, contamination, and unpredictable wound geometry complicated initial clinical assessment, often limiting the ability to determine the full extent of injury until imaging or surgical exploration was performed.

Contamination remained a defining clinical feature in both datasets [15]. The combination of environmental debris, organic matter, and boar-associated oral microbiota contributed to a high risk of infection [4,34,55].

Physiological responses varied depending on the depth and location of injury. Tachycardia, hypotension, and pallor are indicators of significant blood loss or evolving shock. Tachypnea may reflect pain, anxiety, or subtle thoracic compromise. Visible bleeding ranges from profuse arterial hemorrhage to slow but persistent venous oozing from deep muscle wounds. The consistent picture presented by both the observational hunters cohort [15] and the systematic review [15] was that wild boar injuries often appear deceptively benign at first glance, yet frequently conceal extensive and potentially life-threatening internal damage.

Table 3 summarizes injury patterns by anatomic region, drawing on data from the observational hunters cohort [15] and the systematic review [15].

### 3.3. Assessment and Initial Management

The structured dataset of the observational hunters cohort [15] contained only limited information on early assessment or prehospital management. Apart from the time to first help, which varied substantially—from within the first hour for many participants to delays of up to six hours—no structured data were collected on first aid measures, initial clinical evaluation, or transport logistics. Because “first help” included both lay assistance and medical contact, the cohort did not distinguish between care provided by hunting companions and interventions by emergency services. Details such as bleeding control, wound inspection, limb immobilization, or early decision-making were not documented. The four supplementary qualitative cases described later complications rather than initial assessment, and therefore did not extend the insight of the dataset into early management. Overall, the observational hunters cohort [15] provided an outline of how quickly help reached the injured hunters, but offered little information on the specific assessments or interventions performed in the early phase of care.

The systematic review [15] provided more information. Multiple reports described individuals arriving at care in profound shock due to unrecognized vascular injury, especially involving the femoral or deep thigh vessels [29,34,50]. In some patients, uncontrolled prehospital bleeding contributed directly to fatal outcomes [5,34,50]. Early hemorrhage control is therefore a universal first principle of management and remains fully consistent with established trauma algorithms [57,58,59]. Furthermore, the systematic review [15] highlighted challenges commonly faced in early assessment: poor lighting, difficult terrain, environmental stressors, and the frequent inability to fully expose or irrigate the wound in the field [4,9,50]. Pain management is essential to allow adequate examination; however, field responders often lack sufficient analgesic resources. Immobility of the affected limb was frequently necessary to prevent further tissue damage, particularly when the depth or direction of wound tracts was unknown [9,34,40].

Assessment must also consider the mechanism of injury. The tusk-inflicted wounds documented in the observational hunters cohort [15] typically followed an upward curving trajectory, which increased the likelihood of concealed involvement of deeper structures. This pattern suggested that any wound near a major vascular bundle, joint line, or body cavity should be approached as potentially complex until proven otherwise. Both evidence sources [15] indicated that even superficial-appearing wounds may conceal deep muscular separation, joint capsule penetration, or neurovascular disruption [4,29,34]. Given these possibilities, field assessment must err on the side of caution. Red-flag findings requiring immediate evacuation include uncontrolled bleeding, diminished distal pulses, altered sensation or motor function, chest deformity or pain, abdominal tenderness, or any suspicion of penetrating thoracoabdominal injury.

The combined evidence [15] underscored the importance of early evacuation to a facility capable of advanced imaging and operative care. Long delays in transfer, as observed in several case reports in the systematic review [15] from remote regions, correlated with infection development, progression of compartment syndrome, or delayed recognition of organ injury [4,34,45]. During transport, repeated assessment of bleeding, perfusion, respiratory status, and level of consciousness is essential. Field responders should avoid deep probing of wounds and should refrain from attempting closure, as this may trap contaminants or compromise subsequent surgical evaluation.

Ultimately, the consistent message from both the observational hunters cohort [15] and the systematic review [15] was that early management must be systematic, cautious, and oriented toward rapid identification of hidden injuries. The combination of forceful penetration, contamination, and unpredictable wound architecture makes wild boar trauma a deceptively complex presentation requiring rigorous initial assessment [37,42,44].

Figure 2 presents a field-oriented management algorithm informed by evidence from both the observational hunters cohort [15] and the systematic review [15].

### 3.4. Diagnostic Imaging

The observational hunters cohort [15] did not contain structured information on the use of diagnostic imaging. The dataset did not specify whether radiographs, ultrasound, or CT were performed, nor did it provide imaging findings or describe how imaging influenced management decisions. Because imaging was not captured as a variable, no conclusions could be drawn from the observational hunters cohort [15] regarding typical diagnostic pathways or the frequency and relevance of imaging in wild boar-related injuries.

The systematic review [15] expanded on the essential role of imaging across a broader range of clinical presentations. In thoracic injuries, radiography and CT identified pneumothorax, hemothorax, pulmonary contusions, and rib fractures [4,23,36]. Subtle chest findings upon physical examination frequently masked profound internal compromise identified only after imaging [4,23,36]. In abdominal trauma, CT was indispensable to detect free air, organ lacerations, mesenteric injury, or retroperitoneal involvement [4,34,50]. In several international cases, initial examination suggested only superficial injuries, yet imaging revealed deep visceral penetration requiring urgent laparotomy [4,34,45]. This reflects the characteristic tusk trajectory, which may produce deceptively small external wounds despite substantial internal injury. The systematic review [15] also highlighted the value of ultrasound scanning, particularly eFAST [60], in resource-limited environments [4,30,36]. Portable ultrasound enabled rapid identification of hemoperitoneum or pneumothorax in remote settings, guiding immediate management decisions while awaiting transfer [4,30,36].

Joint-related injuries also benefit from imaging. In reports of hand or lower-extremity wounds with suspected joint penetration, CT or radiography was critical in determining whether joint capsules were violated [4,24,44]. Tendon injuries require careful evaluation with ultrasound or MRI, though the latter is rarely performed acutely. Foreign body detection is another important imaging indication. Multiple cases in the systematic review [15] described retained tusk fragments or embedded environmental debris that were only detected after imaging [4,23,45].

Across both evidence sources [15], imaging was consistently shown to reduce the risk of missed injury, refine operative planning, and improve patient outcomes. The complexity and variability of tusk-inflicted wound trajectories make advanced imaging not just beneficial but often essential [30,36,45].

Table 4 presents the differential diagnosis and associated imaging indications, informed by data from the observational hunters cohort [15] and the systematic review [15].

### 3.5. Operative Management

Operative treatment in the observational hunters cohort [15] was closely aligned with the predefined injury categories. All open injuries requiring inpatient care underwent surgery, whereas outpatient open injuries often received operative wound management without admission. Closed injuries were generally treated conservatively. The dataset did not include details about the surgical procedures themselves, such as the extent of debridement, wound exploration, or closure strategy. Therefore, it was not possible to characterize typical operative findings or techniques based on the observational hunters cohort [15] alone. Four participants provided qualitative descriptions of later complications that required revision surgery—such as infection, necrosis, sepsis, compartment syndrome, or removal of a retained foreign body—but these narratives reflect individual cases and cannot be generalized. Overall, the observational hunters cohort [15] documented whether surgery occurred, but not the specific operative approaches or intraoperative findings.

The systematic review [15] highlighted the importance of aggressive and early operative management. Numerous international cases described severe infections and functional impairments arising when initial debridement was insufficient or when wound closure occurred prematurely [4,36,44]. This is likely related to the combination of deep penetrating wound tracts, extensive contamination, and irregular tissue disruption caused by tusk movement during the attack. Tendon injuries, nerve lacerations, and vascular disruptions were frequently identified only during surgical exploration [4,24,29]. Thoracic injuries occasionally required chest tube placement, removal of foreign bodies, or even thoracotomy [4,23,36]. Abdominal injuries mandated exploratory laparotomy, with several cases involving the repair of bowel perforations, control of mesenteric bleeding, or management of organ injury [4,40,45]. In partially healed or delayed-presenting cases, surgeons sometimes encountered necrotic tissue, abscess formation, or advanced soft-tissue infection requiring extensive secondary debridement [4,44,45].

Complex extremity injuries often involved multiple staged procedures, particularly when large soft-tissue defects required flap coverage or grafting [4,34,44]. Joint involvement posed additional challenges, as penetrating joint trauma carries a high risk of septic arthritis. Washout, synovectomy, and prolonged antibiotic therapy were frequently necessary [4,24,44]. The evidence also supports avoiding overly conservative surgical approaches. Minimal debridement or failure to explore the full wound tract increased the likelihood of complications and prolonged recovery [4,36,44].

Across the systematic review [15], operative principles converged on several core themes: follow the wound tract to its deepest point; remove all devitalized and contaminated tissue; irrigate extensively; avoid early closure; reassess frequently; and coordinate multidisciplinary care when complex anatomical regions are involved [4,34,45].

### 3.6. Infection Prophylaxis and Antibiotic Management

The structured dataset of the observational hunters cohort [15] did not include information on infection prophylaxis, antibiotic use, or contamination characteristics. It therefore did not document whether antibiotics were administered routinely, which agents were used, or how long therapy was continued. Among the four participants who provided extended qualitative accounts, postoperative infections and wound complications were described, and these cases required additional surgical management. While these narratives demonstrate that infections occurred in individual cases, they do not allow conclusions about the overall frequency of infection or typical prophylactic strategies in the observational hunters cohort [15]. In summary, the dataset recorded treatment pathways and outcomes, but offered no systematic information on antibiotic management or infection prevention practices.

The systematic review [15] documented a broad range of bacterial pathogens associated with wild boar injuries. Identified organisms include *Pasteurella* species, *Actinobacillus* species, *Streptococcus* species, *Staphylococcus* species, anaerobes, and various components of soil and environmental microbiota [4,34,36,40,44,45]. Several infections progressed to severe soft-tissue infection, septic arthritis, or osteomyelitis [4,24,44]. Necrotizing soft-tissue infections were reported in patients in whom initial wound care was inadequate or in patients who presented late [4,36,44]. Deep infections involving tendon sheaths or closed joint spaces required repeated surgical washouts and prolonged intravenous antibiotic therapy [4,24,44].

The systematic review [15] also emphasized the need for antibiotic regimens that provide broad coverage of both aerobic and anaerobic bacteria. Amoxicillin–clavulanate was commonly used given its wide spectrum [4], but alternative regimens may be required based on allergy profiles or local resistance patterns. For uncomplicated open wounds with substantial contamination but no deep structural involvement, short-course oral antibiotic therapy may be sufficient following adequate surgical debridement. In more severe injuries involving deep tissue destruction, joint penetration, delayed presentation, or systemic infection, initial intravenous broad-spectrum antibiotic therapy followed by culture-guided adjustment may be appropriate. The typical treatment duration reported in the literature ranged from several days for uncomplicated prophylaxis to multiple weeks in patients who developed septic arthritis, osteomyelitis, or extensive soft-tissue infection [4,24,37,43,44]. In severe or life-threatening cases, combinations of agents targeting Gram-negative rods, Gram-positive organisms, and anaerobes are advisable. Duration of therapy varied based on wound severity, operative findings, and contamination level [4,37,43]. In any case, aggressive antibiotic therapy significantly reduces the likelihood of infection-related complications.

Table 5 summarizes empiric antibiotic considerations based on injury severity, contamination burden, and anatomical involvement as described in the studies contained in the systematic review [15].

### 3.7. Rabies and Tetanus Management

Rabies and tetanus prevention are integral parts of early management in wild boar trauma. The observational hunters cohort [15] did not collect data on rabies or tetanus prophylaxis. The systematic review [15] presented a heterogeneous picture of the risk of rabies. In countries where wildlife rabies remains endemic, postexposure rabies prophylaxis was frequently administered after wild boar injuries, particularly when deep wounds or bite-associated mechanisms were involved [4,44,45]. Several reports described administration of both rabies vaccine and rabies immunoglobulin when the rabies status of the wild boar could not be confirmed [4,36,44]. Conversely, in many European and East Asian regions where wild boars do not serve as rabies reservoirs, routine rabies prophylaxis was rarely indicated [4,7,23].

The consistent recommendation is that rabies prophylaxis should be determined by regional epidemiology rather than the species involved. In contrast, tetanus prophylaxis should be provided universally for all open wild boar injuries given the high contamination burden and the severe consequences of tetanus infection. Vaccination status should be assessed early, and booster vaccination or tetanus immunoglobulin should be administered according to standard regional protocols when indicated.

### 3.8. Complications

Complications associated with wild boar trauma are varied and may significantly impact recovery and long-term function. The dataset of the observational hunters cohort [15] did not document specific medical complications such as infection rates, bleeding problems, neurovascular deficits, or other acute or delayed sequelae. It reported only overall healing outcomes and whether persistent functional impairment remained after recovery. Complete healing without lasting impairment was common among participants with closed injuries (22 of 23; 95.7%) and outpatient open injuries (45 of 46; 97.8%), while 8 of 32 (25%) participants with inpatient open injuries reported persistent functional limitations. These residual limitations reflect the greater severity of injuries requiring hospital admission, although the dataset did not specify the underlying causes. Four participants provided extended qualitative accounts of postoperative complications—including delayed infections, wound necrosis, sepsis, compartment syndrome, and a retained foreign body requiring removal. These examples illustrate the potential range of complications but cannot be generalized to the full observational hunters cohort [15]. Overall, the structured data allow conclusions only about final functional outcomes, not the specific complications that occurred during the treatment course.

The systematic review [15] described a broad and often severe range of complications. Deep soft-tissue infections, necrotizing fasciitis, septic arthritis, and osteomyelitis were well documented [4,24,45]. Vascular complications included pseudoaneurysms, delayed hemorrhage, and thrombosis [4,34,49]. Nerve injuries ranged from transient palsies to complete transection requiring surgical repair [4,34,46]. In abdominal and thoracic injuries, delayed recognition of visceral damage led to peritonitis, empyema, overwhelming infection, or systemic sepsis [4,23,45]. Several fatal cases resulted from massive hemorrhage, delayed presentations, or missed internal injuries [41,45,50]. Chronic pain, limited joint mobility, and functional impairment were common in survivors of complex extremity injuries [4,44,52]. Taken together, the evidence highlights that complications can arise even when initial presentation appears mild, particularly when deceptively small wounds conceal deeper tissue damage [9,35,37].

Both the observational hunters cohort [15] and the systematic review [15] emphasized that early, thorough management reduces but does not eliminate the risk of complications. Ongoing monitoring, multidisciplinary care, and patient education regarding warning signs of infection or delayed vascular compromise are essential components of management.

## 4. Discussion

The combined evidence from the previously established observational hunters cohort [15] and the international literature summarized in the systematic review [15] demonstrates that wild boar-related trauma constitutes a distinct and often complex clinical challenge. Each evidence source contributes different types of information. The structured observational hunters cohort [15] provided consistent epidemiologic data on the circumstances of injury, the characteristics of affected hunters, the treatment settings, and the overall healing outcomes. It established that most injuries occurred during daytime tracking operations, predominantly involved experienced hunters, and were frequently caused by male animals [15]. The observational hunters cohort [15] also documented the time to first help and the subsequent treatment pathway, although it did not contain systematic information on wound morphology, contamination, imaging findings, or intraoperative details.

The systematic review [15] complemented these structured observations by providing detailed clinical and forensic descriptions across a wide range of environments, including agricultural, wilderness, peri-urban, and subsistence contexts. Despite geographical and contextual heterogeneity, the reviewed cases revealed recurring clinical patterns: deep and unpredictable wound trajectories, extensive soft-tissue disruption, heavy environmental contamination, and potentially severe neurovascular or visceral injury even when the external wound appeared deceptively small. Thoracoabdominal injuries, although less common, were associated with disproportionate morbidity and mortality. These reports highlight the need for early imaging, aggressive debridement, staged wound management, and broad-spectrum antibiotic therapy.

Several themes emerged when the two evidence streams were integrated. First, the structured observational hunters cohort [15] defined who was injured and how injuries were managed, whereas the systematic review [15] clarified how severe such injuries can be and what hidden risks must be anticipated. Second, the consistency of injury mechanisms across global case reports suggests that certain management principles—such as prioritizing hemorrhage control, maintaining a high index of suspicion for deep structural involvement, and avoiding premature wound closure—are broadly applicable. Third, thoracoabdominal and major vascular injuries, though infrequent, require rapid escalation to advanced care. Fourth, increasing wild boar populations in many regions may increase opportunities for human–animal encounters, reinforcing the need for public-health measures and clinical preparedness.

Overall, integrating the structured data of the observational hunters cohort [15] with the diverse international experience summarized in the systematic review [15] enables a comprehensive and practical approach to assessment and management, particularly in remote or resource-limited settings where these rare but potentially life-threatening injuries may occur. At the same time, these recommendations should be interpreted within the limitations of the available evidence, which is largely based on observational data, case reports, and heterogeneous clinical descriptions rather than formal guideline-level evidence.

## 5. Recommendations/Management Framework

The development of these clinical recommendations for wild boar-related trauma relied on integrating the structured findings of the observational hunters cohort with the diverse international experiences summarized in the systematic review [15]. Although the overall incidence of these injuries is low, their potential severity requires a systematic and well-defined management approach. These recommendations represent an evidence-informed clinical framework rather than a formal guideline developed through consensus methodology.

Early clinical priorities emphasize airway, breathing, and circulation. Hemorrhage control is critical, as uncontrolled bleeding may be a major contributor to morbidity and mortality. Direct pressure should be applied immediately, followed by hemostatic dressings or tourniquets when necessary. These measures should be maintained during transport and reassessed periodically.

Imaging should be performed early and tailored to the suspected injury. Plain radiographs, ultrasound, and CT are essential for evaluating wound trajectories, detecting fractures, identifying foreign bodies, and assessing for thoracoabdominal involvement. In resource-limited settings, portable ultrasound may be the only available modality and can guide immediate management decisions.

Operative management should be proactive and extensive. Surgical exploration must follow the wound tract to its deepest point, exposing all areas of tissue injury. Wide debridement and thorough irrigation are essential because of heavy contamination. Primary closure is rarely appropriate initially, and staged procedures with delayed closure or secondary intention healing are often required.

Infection prophylaxis must begin early, ideally at first clinical contact. Broad-spectrum antibiotics should target both aerobic and anaerobic organisms. The duration of therapy depends on wound severity, contamination level, operative findings, and the presence of infection or joint involvement. Tetanus prophylaxis is universally required considering the contamination burden. Rabies prophylaxis must be based on regional epidemiology rather than species-specific assumptions.

Evacuation decisions must prioritize early transfer for patients with suspected deep structural injury, neurovascular compromise, thoracoabdominal involvement, or uncontrolled bleeding. Field responders should avoid deep probing of wounds or attempting closure. During transfer, continuous monitoring of airway, breathing, and circulation is essential.

Together, these recommendations form a cohesive management framework based on consistent injury patterns and clinical needs demonstrated across structured observational and international evidence. Table 6 summarizes these key recommendations.

## 6. Conclusions

Wild boar-related trauma represents a rare but clinically serious subset of wilderness and rural injuries that can result in deep contamination, complex soft-tissue damage and, in select cases, life-threatening complications. The combined use of a structured observational hunters cohort [15] and a comprehensive systematic review [15] provides, for the first time, an integrated evidence base from which to derive practical, field-oriented recommendations for assessment, early management, and definitive care.

As seen in other uncommon but high-risk trauma scenarios, most notably explosion injuries, for which detailed clinical guidelines have recently been published [11,12], the development of standardized guidance is particularly valuable when provider experience is inherently limited. Although the injury mechanisms differ completely, the comparison underscores a shared structural need: clinicians must be able to rely on reproducible, evidence-informed algorithms when confronted with rare events that nevertheless demand rapid, high-consequence decisions.

Similarly, existing guidelines from the Wilderness Medical Society—including those addressing drowning management [13] and the medical oversight of search-and-rescue operations [14]—illustrate the broader importance of formalized approaches to care in remote, resource-limited, and operationally constrained environments. The present study aligns with this tradition by providing structured, evidence-informed recommendations designed specifically for wild boar-related trauma encountered in wilderness or austere contexts.

By synthesizing epidemiologic insights, detailed case descriptions, and global clinical experience, this study aims to support clinicians, wilderness responders, and prehospital providers who may face these rare but potentially severe injuries. Standardized, context-appropriate management principles may improve patient outcomes, reduce preventable complications, and promote consistent clinical decision-making across diverse care environments.

## Figures and Tables

**Figure 1:**
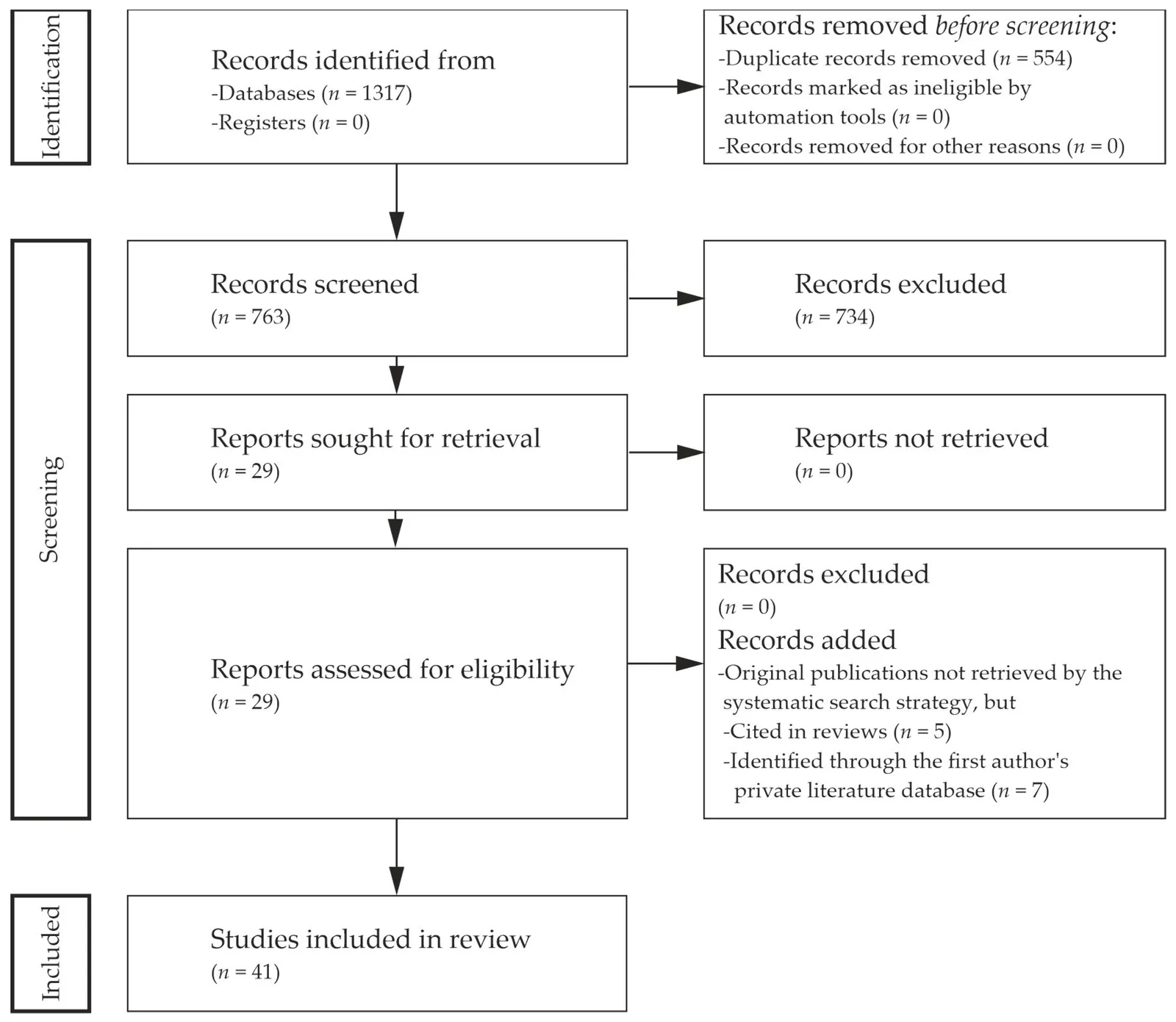
Flow chart of the literature search performed according to the 2020 PRISMA (Preferred Reporting Items for Systematic Reviews and Meta-Analyses) guidelines [16] on 12 February 2026 (taken from [15]).

**Figure 2:**
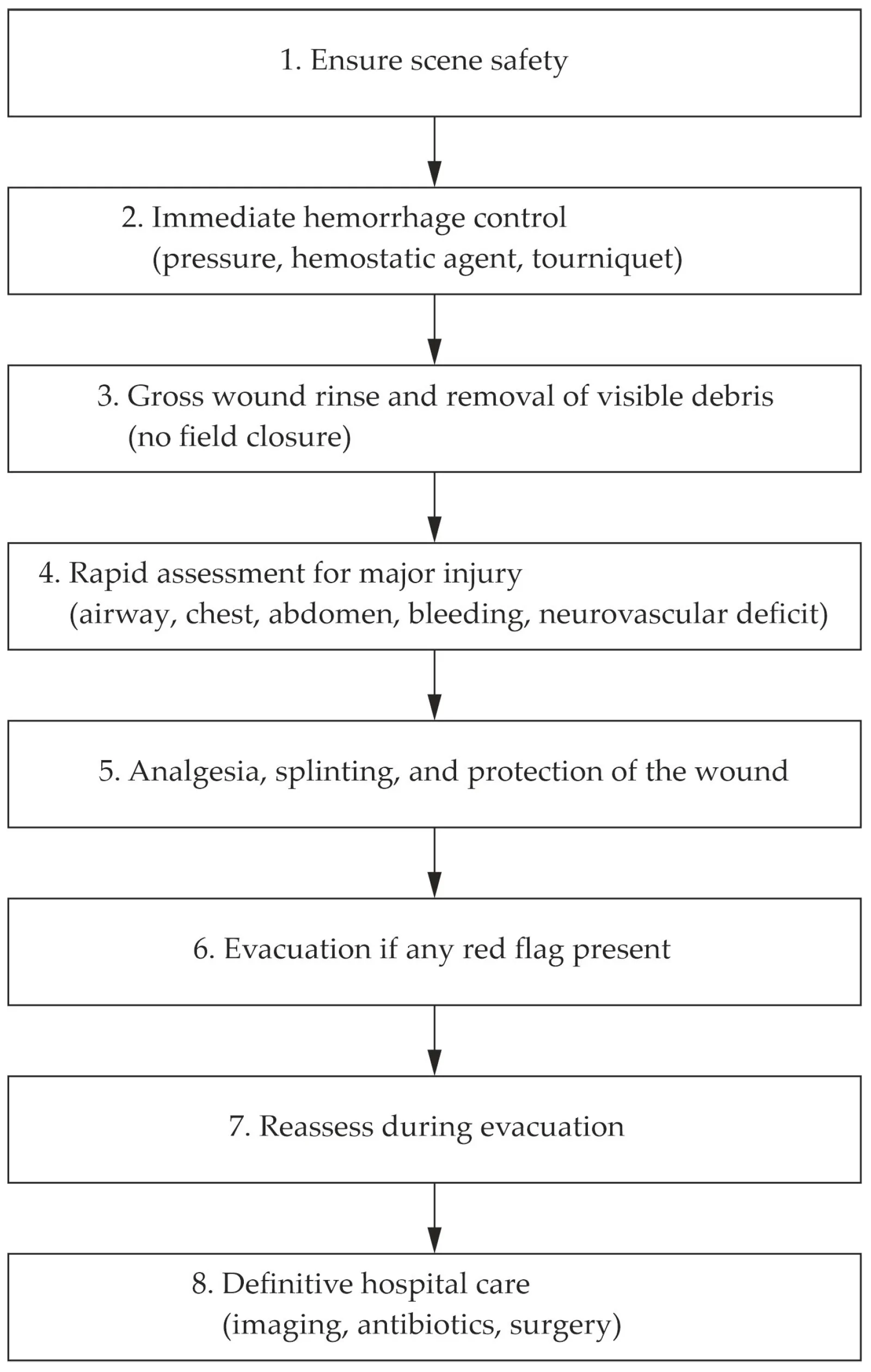
Field-oriented algorithm summarizing recommended assessment and management steps for wild boar-related trauma.

**Table 1:** Terms used in the systematic literature search performed in accordance with the 2020 PRISMA (Preferred Reporting Items for Systematic Reviews and Meta-Analyses) guidelines [16] (taken from [15]). Abbreviations: NR, number of records retrieved; S, sum of records retrieved.

Search No.	PubMed Search	NR	Ovid/Embase Search	NR	
1	("wild boar"[Title/Abstract] OR "Sus scrofa"[Title/Abstract]) AND (injur*[Title/Abstract] OR attack*[Title/Abstract] OR trauma*[Title/Abstract] OR bite*[Title/Abstract]) AND (human*[Title/Abstract] OR patient*[Title/Abstract])	100	((wild boar OR *Sus scrofa*).ti,ab.) AND (injur*.ti,ab. OR attack*.ti,ab. OR trauma*.ti,ab. OR bite*.ti,ab.) AND (human*.ti,ab. OR patient*.ti,ab.)	140	
2	(("pig"[Title/Abstract] OR "boar"[Title/Abstract]) AND (bite*[Title/Abstract])) AND (infection[Title/Abstract] OR injur*[Title/Abstract] OR trauma*[Title/Abstract])	83	((pig OR boar).ti,ab. AND bite*.ti,ab.) AND (infection.ti,ab. OR injur*.ti,ab. OR trauma*.ti,ab.)	86	
3	("animal attack"[Title/Abstract]) AND (boar[Title/Abstract] OR pig[Title/Abstract])	8	(animal attack).ti,ab. AND (boar OR pig).ti,ab.	3	
4	wild boar case report	336	(wild boar).ti,ab. AND (case report OR case study).ti,ab.	36	
5	wild boar attack	104	(wild boar AND attack*).ti,ab.	38	
6	(("Wild boar"[tiab] OR "Sus scrofa"[tiab]) AND ("Wound"[tiab] OR "Injury"[tiab] OR "Attack"[tiab]))	147	((wild boar OR *Sus scrofa*).ti,ab.) AND ((wound OR injury OR attack).ti,ab.)	214	Also used in [5]
7	("wild" OR "feral") AND ("pig" OR "boar") AND ("attack") AND ("fatal" OR "died" or "killed")	8	(wild OR feral).ti,ab. AND (pig OR boar).ti,ab. AND (attack*).ti,ab. AND (fatal* OR died OR kill*).ti,ab.	14	Also used in [6]
S		786		531	

**Table 2:** Condensed overview of evidence included in the systematic review.

Evidence Category	Number of Studies	Typical Setting	Predominant Injury Patterns	Main Contribution to This Study
Case reports	23	Hunting, rural, agricultural, peri-urban encounters	Deep penetrating extremity wounds, thoracic injuries, joint penetration, severe contamination	Detailed clinical descriptions of injury morphology, diagnostic pitfalls, imaging findings, and operative management
Case series/small clinical series	5	Rural/agricultural settings, hunting injuries	Recurrent lower-extremity trauma, soft-tissue destruction, infection	Demonstrate recurring treatment patterns and complication risks
Forensic reports/fatal case analyses	4	Fatal attacks, wilderness/rural settings	Major vascular injury, exsanguination, thoracoabdominal trauma	Highlight mortality mechanisms and importance of hemorrhage control
Retrospective/epidemiologic/registry studies	14	Population-based datasets, wildlife registries, trauma databases	Injury epidemiology, exposure patterns, healthcare utilization	Provide epidemiologic context and identify risk factors for human–wild boar encounters
General wildlife/ecological reviews	2	Broad wildlife–human conflict settings	Mixed injury mechanisms	Provide prevention context and broader wildlife management implications

**Table 3:** Summary of injury patterns by anatomic region based on the observational hunters cohort [15] and the systematic review [15].

Anatomic Region	Primary Evidence [15]	Additional Findings [15]	Clinical Implications
Lower extremities	Most common injury site; deep penetrating wounds; heavy contamination	Consistently reported internationally with long wound tracts	High hemorrhage risk; mandatory imaging and exploration
Groin/pelvis	Injuries near femoral vessels; soft-tissue avulsion	Additional reports of vascular penetration and genital trauma	Rapid hemorrhage control; vascular assessment essential
Abdomen	Occasional upward penetration	Multiple reports of peritoneal violation and visceral injury	Exploratory laparotomy often required
Thorax	Rare in cohort	Reports of pneumothorax, hemothorax, and rib fractures	Requires immediate imaging
Upper extremities	Mainly defensive wounds	Deep-space infections and tendon injuries	High infection risk
Head/neck	Rare	Severe and fatal cases reported	Airway priority; rapid transfer
Neurovascular structures	Occasional involvement	Cases of nerve palsy and arterial injury	Full neurovascular examination essential

**Table 4:** Differential diagnoses and corresponding imaging indications for common wild boar-related injury presentations.

Presentation	Differential Diagnoses	Imaging
Lower-extremity wound	Vascular injury, occult fracture	Doppler US, X-ray, CT angiography
Groin injury	Femoral vessel penetration	CT angiography
Abdominal penetration	Bowel perforation, organ injury	CT with contrast
Chest symptoms	Pneumothorax, hemothorax	X-ray, eFAST, CT
Neurologic deficit	Nerve injury, compartment syndrome	MRI
Joint penetration	Septic arthritis	X-ray, CT, operative assessment

**Table 5:** Empiric antibiotic considerations in wild boar-related trauma. Note: Antibiotic selection and duration should always be individualized based on contamination burden, operative findings, microbiological testing, local resistance patterns, and clinical response.

Clinical Scenario	Typical Microbiological Concern	Suggested Initial Approach	Literature Basis
Superficial contaminated wound	Mixed environmental contamination	Early prophylactic antibiotics after adequate wound care may be considered	[4,36,40]
Deep soft-tissue injury	Polymicrobial contamination	Broad-spectrum coverage should be considered	[4,34,44]
Joint penetration	Septic arthritis risk	Operative washout + prolonged antibiotic therapy may be required	[24,44]
Bone involvement	Osteomyelitis risk	Culture-guided prolonged therapy may be required	[24,43,44]
Necrotizing infection/sepsis	Severe polymicrobial infection	Urgent surgery + broad-spectrum IV antibiotics	[36,44,45]

**Table 6:** Summary of key recommendations for the assessment and management of wild boar-related trauma.

Domain	Recommendation
Early assessment	High suspicion for deep injury
Hemorrhage control	Direct pressure, hemostatic agents, tourniquet
Imaging	X-ray, ultrasound, CT
Surgical care	Thorough exploration, delayed closure
Antibiotics	Early broad-spectrum therapy
Tetanus/rabies	Universal tetanus; rabies per region
Disposition	Early evacuation for major injury

## Data Availability

The anonymized datasets generated and analyzed during this study are available from the corresponding author upon reasonable request and in compliance with institutional and data protection regulations.

## Appendix A

**Table A1:** Overview of all studies included in the systematic review [15] that inform this guideline. Abbreviations: Y, year of publication; C/R, country/region; N, number of human cases, WB, wild boar/wild pig (*Sus scrofa*); DP, domestic pig; WB/DP, wild–domestic hybrid (“javaporco”, etc.).

Study	Y	C/R	Study Type	N	Animal Type	Main Content/Relevance
[17]	2025	USA (central Florida)	Case report	1	WB	Pediatric knee arthrotomy from WB tusk during hunting; intra-articular air on imaging; required urgent irrigation/debridement. Cultures grew Stenotrophomonas maltophilia and *Aspergillus* spp.; recovered fully after targeted therapy.
[18]	2025	Japan	Case report	1	WB	Penetrating thoracic injury with pneumothorax; CT-based diagnosis and chest tube placement; good recovery.
[19]	2024	Nepal	Case report	1	WB	Penetrating lower-extremity WB injury in a rural setting; deep soft-tissue laceration with significant contamination; treated with debridement, irrigation, and antibiotics; uneventful recovery.
[20]	2024	Japan	Case report	1	WB	Penetrating upper-body WB injury in suburban environment; deep soft-tissue trauma requiring surgical debridement; full recovery.
[21]	2024	Turkey	Case report	1	WB	Lower-extremity WB laceration with extensive contamination; operative irrigation/debridement and antibiotics; uncomplicated outcome.
[4]	2024	Spain	Case series/institutional protocol revision	Several	WB	Trauma service experience with WB extremity injuries; emphasizes standardized debridement protocol and broad-spectrum antibiotics.
[22]	2023	South Korea	Case report	1	WB	Lower-limb WB bite/laceration; surgically treated with irrigation/debridement and antibiotics; favorable recovery.
[23]	2023	Japan	Case report	1	WB	Chest/upper-body trauma from WB; emphasizes need for imaging and monitoring even in apparently stable patients.
[24]	2023	Sri Lanka	Case series	Several	WB	Series of WB attacks upon agricultural workers; includes severe perineal, thigh, and abdominal injuries with evisceration; describes surgery, tetanus, and rabies prophylaxis.
[25]	2023	Turkey	Case report	1	WB	Peroneal nerve injury from deep calf laceration; required neurolysis; persistent foot drop; highlights neurovascular risk.
[26]	2022	Turkey	Case report	1	WB	Lower-extremity boar wound; illustrates management and need for surgical debridement and antibiotics.
[27]	2022	Turkey	Case report	1	WB	Penetrating knee joint boar injury with joint involvement; orthopedic washout and infection prevention.
[28]	2021	India	Small series/retrospective	Several	WB/DP (mixed context)	Describes patterns of pig-related wounds (mostly lower-limb), mechanisms, and outcomes; provides additional Indian data on severity and contamination.
[29]	2021	Oman	Retrospective review of animal-related injuries	Subset	WB, among other species	Describes patterns of wildlife trauma; WB cases characterized by lower-limb soft-tissue injuries requiring operative care.
[30]	2021	India	Animal-attack review	Subset	WB, among others	Provides broader context of wildlife injuries in India; WB attacks form a small but severe portion.
[31]	2020	Europe	Spatial/epidemiologic study	N/A	Wildlife, including WB	Spatial analysis of wildlife-related injuries and conflicts; relevant for prevention and risk mapping in regions with WB.
[32]	2018	Global	Review of wildlife trauma	Mixed	Various wild animals	Broad review of animal-related trauma; WB injuries are one component; used mainly for general trauma context, not specific case data.
[33]	2018	Japan	Case report	1	WB	Penetrating chest/rib fracture/pneumothorax; demonstrates thoracic risk even with small skin wounds.
[34]	2018	Japan	Case report (WEM)	1	WB	Complex attack: femoral artery and sciatic nerve injury, anorectal injury, pelvic fractures, open pneumothorax; prolonged, staged surgical management and complications.
[35]	2018	Brazil (national)	Retrospective analysis of human rabies treatment forms	309 WB/WB–DP attacks	WB and WB/DP (“javaporcos”)	Nationwide Brazilian dataset: 309 attacks (271 WB, 38 hybrids); mainly deep single bites to lower limbs; anti-rabies serum + vaccine most commonly indicated; shows expanding WB invasion and health-system response.
[36]	2018	India	Case report	1	WB	Penetrating chest injury from WB with pneumothorax and subcutaneous emphysema; diagnosed via ultrasound and CT; treated with tube thoracostomy.
[37]	2017	Japan	Case report	1	WB	WB bite wounds to lower extremity; significant contamination; wound infection requiring re-debridement.
[38]	2017	Nepal (Chitwan NP)	10-year wildlife conflict registry	38 WB attacks	WB	Wildlife attacks on people in buffer zone; 38 WB-related events with pattern of lower-limb injuries and risk factors (time of day, activity).
[39]	2017	India	6-year autopsy series of animal-attack deaths	Subset	Mixed, including WB	Describes human fatalities caused by animal attacks; WB attacks are a subset, emphasizing that WBs can be a cause of fatal trauma.
[40]	2016	India	Case report	1	WB	Perineal penetrating injury with recto-vesico-cutaneous fistula after WB attack; complex pelvic reconstruction and fistula repair.
[41]	2015	India	Forensic case report	1	WB	Fatal penetrating thigh wound from WB tusk with femoral vessel injury; illustrates exsanguinating potential of lower-limb tusk injuries.
[42]	2015	India	Forensic case report	1	WB	Fatal tusk injuries to thigh with transection of femoral vessels and nerve; provides detailed autopsy description of hemorrhagic death.
[43]	2014	Croatia	Case report	1	WB	Single WB-inflicted lower-extremity bite wounds in hunter; irrigation, debridement, primary closure; antibiotics; no complications.
[44]	2013	Croatia	Retrospective series of hunter injuries	Several	Mixed game, including WB	Patterns of game-animal attacks on hunters; WB a prominent species; lower-limb soft-tissue injuries dominate; most require surgical management.
[45]	2011	India	Case report	1	WB	Facial/maxillofacial injury caused by WB; deep soft-tissue laceration; emphasizes need for surgical debridement and layered closure.
[46]	2011	Turkey	Case report	1	WB	Severe thigh laceration from WB attack; surgically treated; underscores contamination risk and need for broad-spectrum antibiotics.
[47]	2010	India	Case report	1	WB	Severe scrotal/testicular laceration by WB; highlights groin vulnerability and reconstructive issues.
[48]	2008	India	Case report	1	WB	Major thigh injury with significant hemorrhage during WB encounter; demonstrates risk of shock and need for emergency surgery.
[49]	2007	Turkey	Case series	3	WB	Wild boar attacks—three rural cases; significant soft-tissue trauma, mostly lower extremities; one case with upper abdomen/chest involvement.
[50]	2006	India	Forensic case report	1	WB	Fatal WB attack with deep groin and thigh penetration and major vascular injury; classic example of exsanguination mechanism.
[51]	1996	France	Single infected-bite case	1	DP	Actinobacillus suis infection after DP bite to knee; highlights unusual pathogens and confirms amoxicillin–clavulanate as good empiric choice.
[52]	1992	Switzerland (WEM)	Case report	1	WB	Septic arthritis of knee after WB attack; mixed Pasteurella multocida and Bacteroides fragilis infection; classic example of deep joint contamination.
[53]	1991	USA	Case report	1	Domestic boar	Severe hand wound with deep-space infection after domestic boar bite; emphasizes need for aggressive debridement and antibiotic therapy.
[54]	1990	USA	Case report (microbiology)	1	Pig (DP)	Hand infection following pig bite; recovery of unusual Flavobacterium group IIb-like organism; part of pathogen spectrum relevant to pig/WB wounds.
[55]	1988	United Kingdom (North Yorkshire)	Case series	7	Swine (DP/boar)	Pig bite injuries and infection; deep posterior-thigh lacerations and other wounds; mixed infections (streptococci, Pasteurella, Bacteroides, etc.); argues for broad-spectrum antibiotics in severe pig bites.
[56]	1988	Papua New Guinea	6-year hospital-based review	15 pig-injury admissions	Mainly DP, some feral pigs	Med J Aust paper: injuries caused by pigs; mixture of abdominal, chest, limb, and scrotal wounds; includes “sucking” chest wound, bilateral pneumothoraces, open fractures, nerve, and tendon injuries; demonstrates severity of pig gorings.
[6]	2023	Global	Extended/updated review	>400 attacks/fatalities	Wild pigs	Expanded analysis of worldwide wild-pig attacks and fatalities; confirms pattern of severe soft-tissue and blunt trauma, with majority of deaths due to hemorrhage.
[3]	2023	France/Europe	Socio-ecologic analysis	N/A	WB	Addresses synurbic WB and human–boar conflicts in peri-urban settings; directly relevant for prevention and public-health messaging.
[2]	2020	Poland/Europe	Ecologic/management paper	N/A	WB	Explores WB population management, hunting pressure, and landscape changes; informs risk reduction discussion.
[7]	2019	Nordic countries/literature-based	Forensic literature review of fatal attacks by large mammals in the Nordic wilderness	3 fatal WB cases extracted from the literature	WB (plus bear, moose, wolf)	Reviews species-specific injury patterns in fatal attacks by brown bear, moose, wild boar, and wolf; WB section synthesizes three Indian fatal boar cases (lower-body tusk penetration, exsanguination, or craniocerebral injury) to help forensic differentiation.
[1]	2015	Europe (general)	Ecologic/population review	N/A	WB	Describes expanding WB populations and implications for human–boar interactions and conflicts; crucial for prevention context.
[5]	2013	Global	Narrative review	412 attacks compiled	Wild pigs (WB + feral)	Comprehensive global review of wild-pig attacks; describes circumstances (hunting, non-hunting), injury patterns (single lacerations, puncture wounds, fractures), and causes of death (mostly exsanguination).
[9]	2004	North America (Yellowstone/US context)	Narrative review of wild mammalian injuries	Not specified (review)	Various wild mammals (not pig-specific)	This publication addresses general patterns of wild animal trauma, field care, wound management, antibiotic choices, rabies, and tetanus guidance; used for general wilderness/animal-attack management principles, not WB case data.
